# Curative-Intended Management of Synchronous Esophageal and Rectal Cancer—A Systematic Literature Review

**DOI:** 10.1007/s12029-025-01170-7

**Published:** 2025-01-13

**Authors:** Georg W. Wurschi, Claus Schneider, Thomas Ernst, Herry Helfritzsch, Jens Nowatschin, Thomas Bitter, Martin Freesmeyer, Klaus Pietschmann, Maximilian Römer

**Affiliations:** 1 Department of Radiotherapy and Radiation Oncology, Jena University Hospital, 07747 Jena, Germany; 2 Clinician Scientist Program, Interdisciplinary Center for Clinical Research (IZKF), Jena University Hospital, 07747 Jena, Germany; 3 Comprehensive Cancer Center Central Germany, Campus Jena, 07747 Jena, Germany; 4 Department of General, Visceral and Vascular Surgery, Jena University Hospital, 07747 Jena, Germany; 5 Klinik Für Innere Medizin II, Hematology/Oncology, Jena University Hospital, 07747 Jena, Germany; 6 Department of General, Visceral and Thoracic Surgery, Thuringia-Clinic Saalfeld Georgius Agricola, 07318 Saalfeld, Germany; 7 Department of Internal Medicine, Thuringia-Clinic Saalfeld Georgius Agricola, 07318 Saalfeld, Germany; 8 Department of Otorhinolaryngology, Jena University Hospital, 07747 Jena, Germany; 9 Clinic of Nuclear Medicine, Jena University Hospital, 07747 Jena, Germany

**Keywords:** Neoadjuvant Treatment, Synchronous Cancer, Rectum, Esophagus

## Abstract

**Purpose:**

Synchronous esophageal (EC) and rectal carcinoma (RC) is a rare and challenging condition, particularly in curative-intended treatment. Especially locally advanced tumors may not be suitable for primary resection and require individual multimodal treatment. This review examines curative-intended management of synchronous EC and RC.

**Material and Methods:**

A systematic literature search across five electronic databases according to the PRISMA guideline was conducted. Individual patient data was analyzed, including two additional cases from our institution.

**Results:**

We identified 9 relevant cases from 1552 results. Additionally, two male patients (62 and 65 years old) from our institution were included. Both received 5-fluorouracil/cisplatin-based chemoradiotherapy (CRT) for EC. Sequential short-course radiation (SCRT) for RC was performed in one patient. After complete response (CR) in both tumors, no consecutive surgery was performed. He underwent resection for local recurrence of RC 11 months later and is currently considered as disease-free (30 months follow-up). The second patient underwent primary resection of RC and had early progression following resection of EC. We found that most patients had advanced EC (8/11), with the majority receiving neoadjuvant (5/11) or definitive treatment (3/11). Locally advanced RC was diagnosed in 5/11 patients, primarily treated with sequential resection. Pyrimidine-based systemic treatment was common. Four relapses and two deaths were reported, but median follow-up was 11 (range 1.5–30) months only.

**Conclusion:**

The review suggests that neoadjuvant multimodal approaches may offer curative potential for synchronous EC and RC, with individualized treatment protocols adapted from single-cancer protocols. Nevertheless, data on long-term outcome is limited.

**Supplementary Information:**

The online version contains supplementary material available at 10.1007/s12029-025-01170-7.

## Introduction

The simultaneous incidence of esophageal cancer (EC) and rectal adenocarcinoma (RC) is a rare condition in the clinical setting, resulting in complex challenges regarding oncological care. Especially in the context of a curative treatment, highly effective approaches for distinct tumors and adequate timing of every measure are required. Optimal diagnosis, treatment strategies, and overall patient management are not well studied. In case of early-stage disease, primary resection might be considered. Nevertheless, a substantial part of EC and RC is discovered in a locally advanced stage due to either the late onset of clinical symptoms (RC) or early tumor-specific lymphatic and vascular spread (EC), for example. Primary resection alone might thus not be realizable due to advanced tumor infiltration or is known to be associated with compromised results in terms of local tumor control. Neoadjuvant chemoradiotherapy (CRT) or neoadjuvant chemotherapy can be considered as standard of care (SOC) for locally advanced disease in EC and RC, respectively [1, 2]. For both esophageal squamous cell carcinoma (ESCC) and esophageal adenocarcinomas (EAC), the CROSS regimen, consisting of neoadjuvant RT (total absorbed dose, TAD 41.4 Gy/single fraction dose, SFD 1.8 Gy) with concomitant carboplatin/paclitaxel, is referred to as current SOC [2, 3]. Considering the high response rates, also definitive CRT, as by the RTOG 85–01 protocol (including cisplatin/5-fluorouracil, 5-FU), is an alternative treatment in curative intent for SCC [4]. Conversely, intensified neoadjuvant protocols for RC (so-called total neoadjuvant therapy (TNT)) are including either consolidation or induction chemotherapy and have demonstrated increased complete response (CR) rates compared to neoadjuvant CRT alone [5–8]. As non-operative management (NOM) in case of CR has been proven to be safe in terms of tumor control [9, 10], it becomes increasingly applied in clinical practice, and these TNT schedules may thus allow for intended organ preservation in selected cases [1]. Multidisciplinary care and, in particular, high-quality imaging are required in order to be able to assess CR non-invasively. Advances in magnetic resonance imaging (MRI), especially the introduction of diffusion-weighted imaging (DWI), and the availability of endorectal ultrasound (REUS) are recently enhancing biopsy-driven response assessment [11–14].

Nevertheless, there is currently no reliable data on outcomes and toxicity of individualized multimodal schedules addressing both neoplasms simultaneously in curative intent. This article comprises a systematic literature search regarding synchronous esophageal and rectal cancer treatment with curative intend, focusing on the role of multimodal therapy. Therein, two patients are reported, who underwent neoadjuvant/definitive CRT in that condition at our cancer center, and their follow-up.

## Material and Methods

We performed a systematic literature review on individual patient reports across multiple electronic databases, including PubMed, Scopus, Cochrane Database, OVID Medline, and Web of Science. The search was conducted from inception until October 2024. The search strategy utilized a combination of keywords and Medical Subject Headings (MeSH) terms related to “esophageal cancer,” “rectal cancer,” and “simultaneous”/ “synchronous” disease and curative-intended management. The complete search string and the search strategy according to PICO (Population, Intervention, Comparison, Outcome) criteria, are provided in the Supplement Table 1.

Data extraction was performed by one reviewer (MR). All characteristics were assessed by two independent reviewers (MR, GW). In case of disagreement, a third reviewer was consulted (KP) and consensus was made by discussion. Only reports including individual patient data were extracted from retrospective studies. The hereby identified cases were analyzed together with two own cases, treated at our cancer center for synchronous EC and RC with curative intend.

All outcomes are reported according to RECIST-criteria [15] and the 8th UICC TNM-classification [16]. Methodological quality of the included reports was assessed as proposed by Murad et al. [17]; a minimum follow-up period of 12 months was considered being adequate for early assessment of outcome. Individual patient data are summarized and analyzed descriptively, using SPSS, version 29 (SPSS Inc., Chicago, IL, USA).

## Case Presentation

### Case 1

**Fig. 1:**
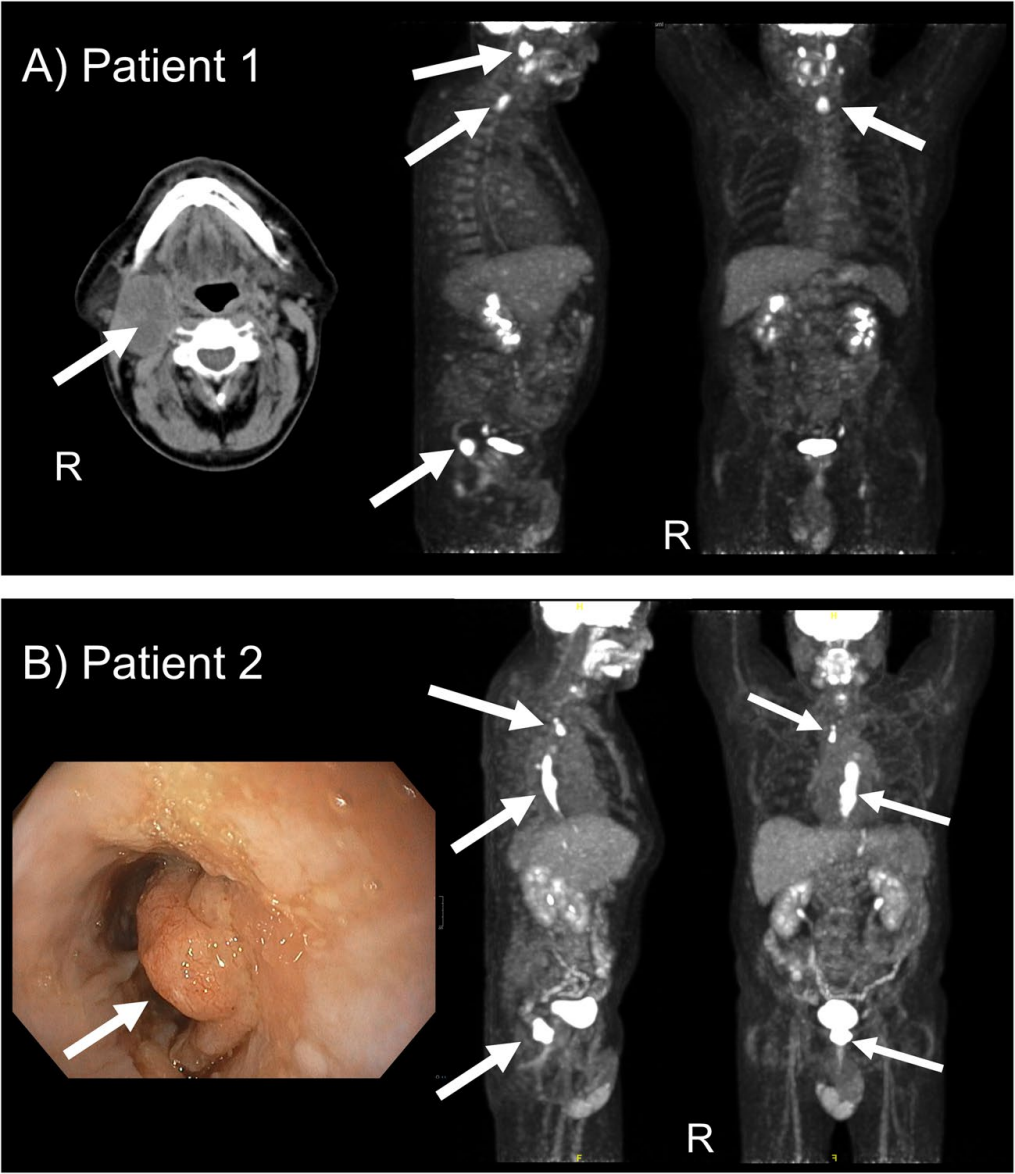
**A** Pretherapeutic staging with FDG-PET/CT revealed cervical esophageal cancer with a cervical metastasis, infiltrating the right parotid gland and a synchronous rectal cancer in patient 1, as illustrated by maximal-intensity projection (MIP) coronal and sagittal views of the FDG-PET (right side). A coincidental prostate cancer was found in this patient but was hardly visible in this MIP window. **B** Patient 2 was diagnosed with synchronous thoracic esophageal cancer and locally advanced rectal cancer, as shown in the coronal and sagittal MIP FDG-PET images. An exemplary image from pretreatment esophagoscopy illustrates the exophytic esophageal cancer. White arrows were placed in (**A**) and (**B**) to highlight localization of disease

A 65-year-old male patient was referred to our cancer center for a 5 × 3 × 2 cm, cystic mass, with infiltration of the right parotid gland and the jugular vein. He underwent partial parotidectomy and extended radical neck dissection (levels I–V). Histopathology revealed a SCC metastasis, G2, p16 negative with positive margin, and no further lymphatic spread (0/21 nodes). As intubation appeared to be difficult due to stenosis, an endoscopy plus endotracheal ultrasound were performed additionally, showing an ulcerating cervical EC with bronchial invasion, which was confirmed histopathologically (ESCC, G3). FDG-PET/CT was carried out for whole body staging (Fig. 1A), confirming hypermetabolic EC and related hypermetabolic parapharyngeal and cervical lymphatic nodes (cT4 cN + cM1). The cervical metastasis was considered to originate from ESCC. Beyond that, a hypermetabolic rectal wall thickening, suspicious for rectal cancer, and unclear metabolic changes within a moderately enlarged prostate were detected. Pelvic MRI and colonoscopy confirmed an exulcerated, non-stenosing RC 8–10 cm from the anal verge and a suspicious regional lymph node. Neither infiltration of mesorectal fascia (CRM-) nor extramural vascular invasion (EMVI-) were detected (cT3 cN1 cM0). As MRI further showed T2 inhomogeneities with corresponding restricted diffusion within the prostate, he underwent subsequent prostate biopsy. A cT1c cN0 cM0 acinous adenocarcinoma, G1, Gleason score 3 + 3 = 6 (9/12 positive specimens) was discovered and classified as intermediate risk tumor, regarding the elevated initial prostate specific membrane antigen (PSA) at 18.7 ng/ml.

**Fig. 2:**
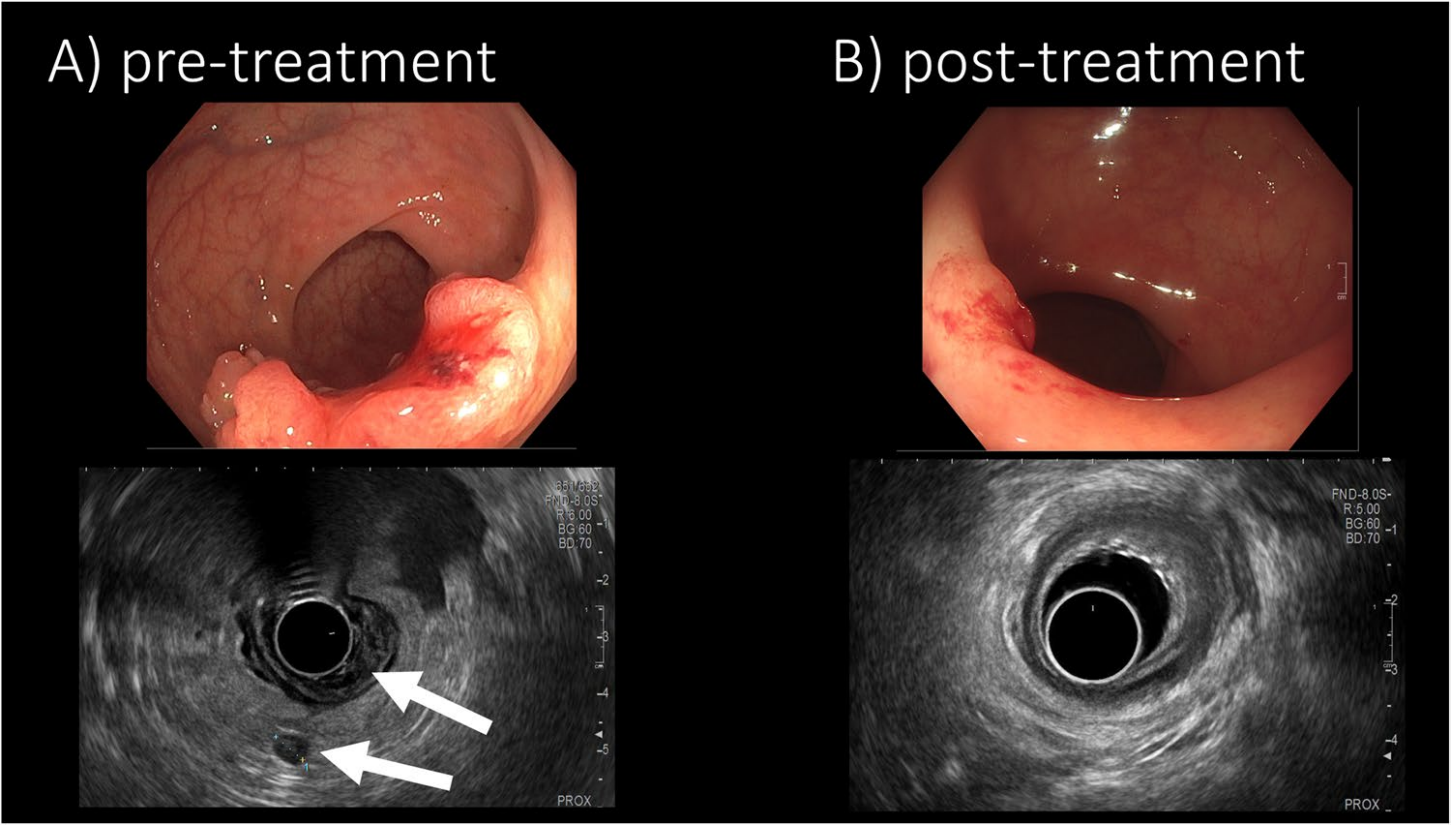
Colonoscopy and endorectal ultrasound (REUS) images of patient 1 before (**A**) and after chemoradiotherapy (**B**), showing a complete response (CR) of an exophytic locally advanced rectal cancer with positive mesorectal node (T3N1, white arrows) after two courses chemotherapy (cisplatin/5-fluorouracil) and sequential short-course radiotherapy (5 × 5 Gy). A discrete scar with telangiectasia was observed and histopathologically evaluated, confirming pathological CR (**B**)

Considering the prognosis of these three synchronous cancers, a prioritized treatment of EC with subsequent RC resection was recommended in our multidisciplinary tumor board review. A definitive CRT for EC (total absorbed dose, TD 50.4 Gy/single fraction dose SFD 1.8 Gy), including a boost on the esophageal mass (TD 56/SFD 2 Gy) and an extended RT-field to account for microscopic residual disease after R1-resection of the parotid metastasis, was performed. He received two cycles concomitant chemotherapy (5-FU 1000 mg/sqm/d d1-4 and cisplatin 20 mg/sqm/d d1-5, qd29) and underwent sequential pelvic short-course RT (TAD 25 Gy/SFD 5 Gy) as neoadjuvant treatment for RC. During this treatment, no severe toxicity (CTCAE °III/ +) was observed. Multimodal response assessment, including CT, pelvic MRI, esophageal/rectal endoscopy plus ultrasound, and biopsy, showed complete response (CR) to both EC and RC (Fig. 2). He then entered close follow-up. PSA remained stable within follow-up, so that no active treatment was initiated. Local recurrence of RC was detected 11 months after treatment. He underwent total mesorectal excision (TME) and experienced anastomotic insufficiency. Currently (30-month follow-up), he is in good general condition with no evidence of disease (NED) for both EC and RC and prostate cancer under active surveillance, respectively.

### Case 2

We report on a 62-year-old male, who was diagnosed with distal high-grade (G3) ESCC for dysphagia and weight loss. FDG-PET/CT showed hypermetabolic local nodes (cT3 cN + cM0) but also revealed a hypermetabolic rectal wall thickening incidentally (Fig. 1B). Pelvic MRI and colonoscopy confirmed a T3 N0, CRM- EMVI-rectal adenocarcinoma, which was located 7–12 cm from the anal verge. He was scheduled for neoadjuvant CRT for EC (TAD 41.4 Gy/SFD 1.8 Gy) with concomitant 5-FU/cisplatin and sequential short-course RT (TAD 25 Gy/SFD 5 Gy). Tolerability of CRT was bad due to repeated bleeding of EC (CTC °IV) and severe pancytopenia (CTC °III) after the first course cisplatin/5-FU, requiring endoscopic intervention and red blood cell transfusion. He did neither receive the second course chemotherapy nor the scheduled neoadjuvant short-course RT for RC for these reasons and reduced general health condition. Low-anterior resection for RC was performed subsequently (pT3 pN0 L1 V1 R0). After reconstitution, he underwent robot-assisted minimally invasive esophagectomy (RAMIE) for EC, revealing partial response, PR, (ypT2 ypN0 L0 V0) but positive margins (R1). He was diagnosed with PD (lymphatic and pulmonal metastasis) 10 months after diagnosis and subsequently developed a painful metastasis of the right ilium. Palliative RT (TAD 36 Gy/SFD 3 Gy) was administered and provided good symptom relief. A palliative immune-checkpoint inhibition (ICI) was performed with nivolumab 240 mg q2w. There is currently (24-month follow-up) NED for RC and SD regarding EC. The patient remains in reduced general condition (ECOG 2) due to late dumping syndrome with repeated complicated hypoglycemia and pneumonitis after ICI.

## Summarized Results of the Literature Research

**Fig. 3:**
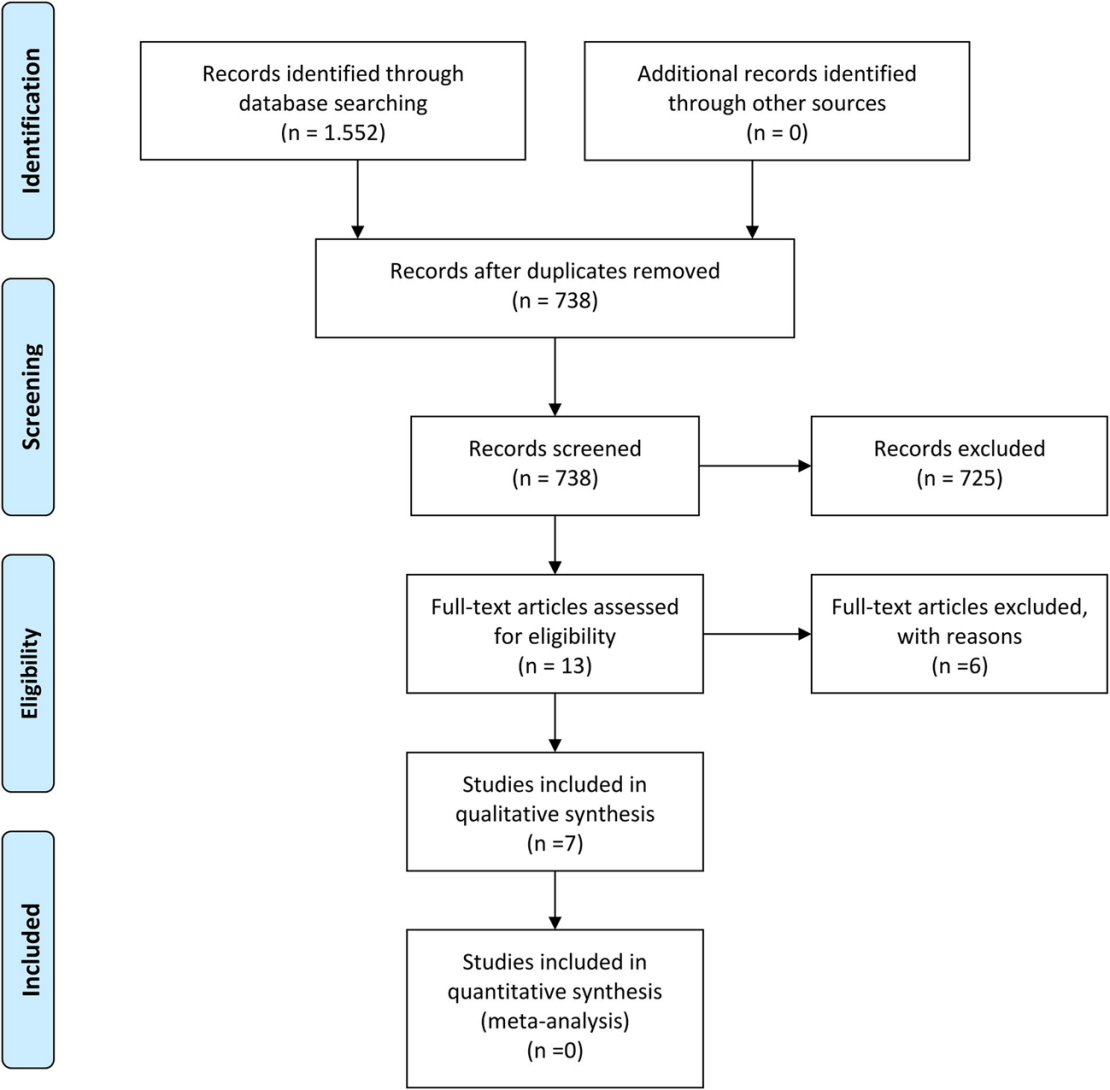
A PRISMA chart, reporting the systematic research workflow applied for this analysis, according to Moher et al. [18]

The systematic search is reported following the PRISMA guideline [18] and revealed 1552 results (Fig. 3). After removal of duplicates, remaining 738 articles were screened for matching titles and abstracts. We found 13 articles eligible for full-text screening. Excluded studies and respective reasons are provided in full in the Supplement Table 2. Finally, a total of 7 publications, reporting on 9 individual patients, was included in further evaluation together with two own cases, reported in this article.

**Fig. 4:**
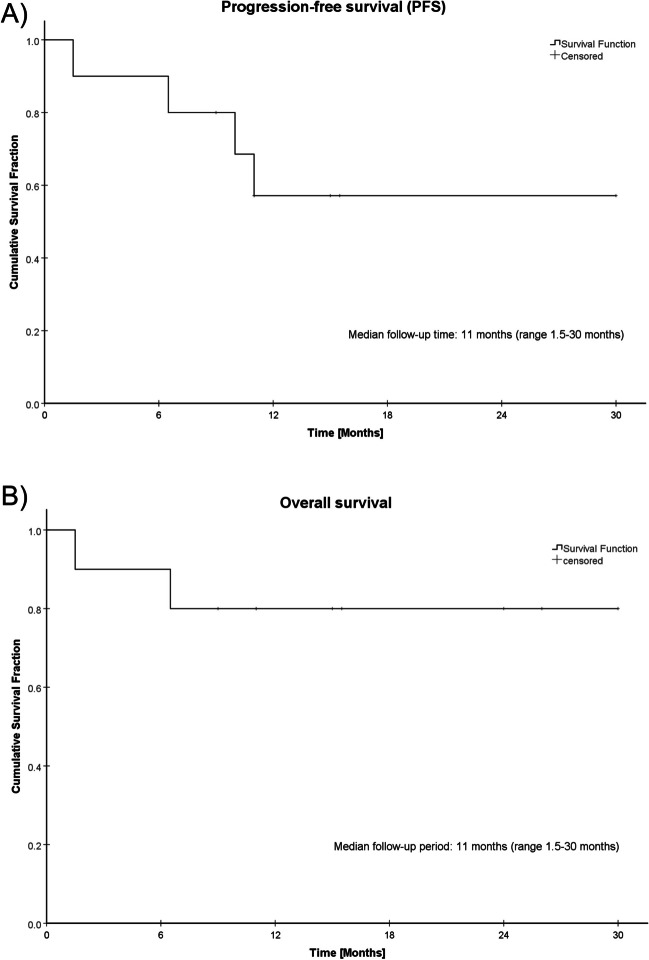
A + B Kaplan–Meier curves of the analyzed patients (*N* = 10, *N* = 1 excluded due to missing data) for **A** overall survival (OS) and **B** progression-free survival (PFS). Median PFS and OS were not reached as the reported median follow-up period was 11 months (range 1.5–30 months) only

The individual tumor characteristics which reported first-line treatment and outcomes are summarized in Table 1. The cohort comprises only men with a median age of 67 years (range 61–77 years). EC was mostly staged as locally advanced in 8/11 patients (72.7%). ESCC was the dominant histological subtype (81.8%). Regarding RC, locally advanced rectal cancer (LARC) was reported in 5/11 patients (45.5%). The treatment was predominantly consisting of multimodal approaches, and a primary resection of both EC and RC was performed in 3/11 patients only (27.3%). All other patients underwent neoadjuvant chemo(radio)therapy or definitive CRT (3/11 patients). Due to stenosis, three patients underwent primary resection of RC, whereas EC was managed prioritized in all other patients. RC was then resected sequentially. We were reporting the only case receiving dedicated neoadjuvant RT for RC and entering NOM after CR. Adjuvant treatment was administered in 5/11 patients (45.5%), including mainly adjuvant chemotherapy (4/11). An overview of the performed treatments and summarized characteristics of the cohort is provided in Table 2. The treatments were mainly performed safely, but, unfortunately, one treatment-related death, related to systemic treatment, were found. Within the reported follow-up period (median 11 months, range 1.5–30 months), one cancer related death occurred (Fig. 4A). Progressive disease was reported in four cases only within this, relatively short, follow-up period (Fig. 4B).

**Table 1:** Overview of clinical characteristics, treatment, and outcomes of patients with simultaneous diagnosis of esophageal cancer and rectal cancer retrieved from the systematic literature research. *AC* adenocarcinoma, *G1-3* tumor grading, *m/f* gender, *EC* esophageal cancer, *RC* rectal cancer, *PR* partial response, *NR* no response, *CR* complete response, *NED* no evidence of disease, *d/n/pCRT* definitive/neoadjuvant/palliative chemoradiotherapy, *n/p/d/adCTx* neoadjuvant/palliative/definitive/adjuvant chemotherapy, *ad/nRT* adjuvant/neoadjuvant radiotherapy, *MSI* microsatellite instable, *MSS* microsatellite stable, *†* death, *PNP* polyneuropathy, *SFD* single fraction dose, *TAD* total absorbed dose, *eoFU* end of follow-up, *seq* sequential, *conc*. concomitant, *n.a.* not available

Case	Author	Country	Age [years] / Sex	Esophageal cancer		Rectal cancer		Treatment	Toxicity‚ (CTCAE v5.0 grade)	Follow-up (months)	Outcomes	Remarks	Methodological quality according to Murad et al. [17]
				TNM Localization	Histology	TNM Localization	Histology						
1	Hayashi et al. 1994 [19]	Japan	67, m	cT4cN + cM0, laryngeal, bronchial + aortic invasion Thoracic esophagus	SCC, G2	cT1, mid-rectum	AC, G2	nCTx (Cisplatin / 5-FU / Vindesine) Hypopharyngeal tumor: mucosa dissection RC: transanal resection EC: esophagectomy adRT: cervical (TAD 45 Gy) and esophageal tumor bed (TAD 60 Gy)	n.a	11	PR (all) after nCTx, EC: pT3pN3pM0 RC: pT1 L1V1, R1 eoFU: NED (all)	Simultaneous hypopharynx tumor (T1)	Limited quality (no data regarding toxicity reported, short follow-up)
2	Yoshii et al. 2019 [20]	Japan	68, m	cT3cN1, middle thoracic esophagus	SCC	cT3 cN1, rectosigmoid	AC	EC: nCRT (TAD 41.4 Gy / SFD 1.8 Gy), conc. FOLFOX × 3 Curative resection (EC + RC)	Esophagitis, °IV encephalitis (5-FU induced)	6.5†	EC + RC: SD after nCRT, EC: R2-resection, early progress eoFU: Death (cancer-related)		Good quality (long follow-up, adequate description of measures and outcomes)
3	Yoshii et al. 2019 [20]	Japan	65, m	cT3cN2, middle thoracic esophagus	SCC	cT3cN0, rectosigmoid	AC	EC: dCRT (TAD 60 Gy / SFD 2 Gy), conc. FOLFOX × 3 dCTx: seq. FOLFOX × 3	none	15.5	CR (RC), SD (EC) eoFU: NED (RC) / SD (EC)		Good quality (long follow-up, adequate description of measures and outcomes)
4	Yoshii et al. 2019 [20]	Japan	77, m	cT2cN1, lower thoracic esophagus	SCC	cT3cN0, upper rectum, stenosing	AC	RC: primary resection (stenosis) EC: dCRT (TAD 60 Gy / SFD 2 Gy), con FOLFOX × 3 adCTx: FOLFOX × 3 + Capecitabine (mono) × 4	°II PNP	15	ER: CR RC: pT3pN1,R0 eoFU: NED (all)	FOLFOX discontinued due to PNP	Good quality (long follow-up, adequate description of measures and outcomes)
5	Imamura et al. 2020 [20]	Japan	74, m	cT2cN0M0, lower thoracic esophagus	SCC	cT3N0M0, upper rectum, stenosing	AC	RC: primary resection (R0) Pancreatic cancer: nCTx Gemcitabine + nab-Paclitaxel × 10; seq. Pancreatoduodenectomy EC: dCRT (TAD 59.4 Gy / SFD 1.8 Gy), cisplatin/5-FU conc adCTX: S1	Portal vein thrombosis	9	RC: pT3N0M0, R0 Pancreatic cancer: ypT1cN0M0 EC: CR eoFU: NED (all)	Additional synchronous pancreatic cancer, cT3cN0cM0	Good quality (long follow-up, adequate description of measures and outcomes)
6	Jena et al. 2024 [21]	India	77, m	n.a	AC, G1	pT3pN2b, G3, rectosigmoid	Signet ring cell AC, G2,	RC: primary resection adCTx: 2 × capecitabine EC: none	Lung collapse postoperative °V intestinal obstruction	1.5†	eoFU: Death (treatment related)	Additional frontal midline meningioma, patient refused primary EC resection	Limited quality (no TNM for EC)
7	Motoori et al. 2001 [22]	Japan	75, m	pT1pN0pM0, middle thoracic esophagus	SCC, G3	pT3pN2M0, upper rectum	AC, G2	RC: primary resection EC: blunt mucosa dissection	none	11	RC: pT3pN2M0 EC: pT1pN0pM0 eoFU: NED (all)	Paraneoplastic Prurigo chronica multiformis	Limited quality (short follow-up)
8	Utsunomiya et al. 2013 [23]	Japan	61, m	TxcN0, middle thoracic esophagus	SCC, G3	cT4cN0cM1	AC, G2	nCTx: FOLFOX-cetuximab × 6 RC: seq. resection + partial hepatectomy (liver metastasis) EC: seq. esophagectomy after reconstitution (+ 5 months) Right lower lobectomy (for later pulmonary metastasis)	Postoperative wound/urinary tract infection, anastomotic insufficiency	26	RC: ypT4ypN0ypM1 EC: ypT1bypN1 PD (RC): pulmonary metastasis eoFU: NED (all)	Successful, curative-intended treatment for oligometastatic synchronous cancer	Good quality (long follow-up, adequate description of measures and outcomes)
9	Kit et al. 2016 [24]	Russia	65, m	cT3cN0 M0, middle thoracic esophagus	SCC, G3	cT3cN0cM0, rectosigmoid	AC, G2	Primary radical resection of all three cancers; small bowel interposition adCTx: CapOx × 6	none	n.a	Complete resection of all three cancers, eoFU: n.a	Additional gastric cancer (AC, G2): cT3cN0cM0	Fair quality (insufficient reporting of toxicity, outcome and follow-up)

**Table 2:** Summarized frequencies of clinical features and performed treatments. As certain patients received both neoadjuvant and adjuvant treatment, total numbers exceed the number of patients (*n* = 11). *UICC* Union Internationale Contre le Cancer

Characteristics/treatment	Number of patients	Percent
Histology		
Esophageal squamous cell cancer (ESCC)	9/11	81.8%
Esophageal adenocarcinoma (EAC)	2/11	18.2%
Rectal adenocarcinoma	11/11	100%
Locally advanced esophageal cancer * UICC Stages II–IVA*	8/11	72.7%
Early esophageal cancer * UICC Stage I*	3/11	27.3%
Locally advanced rectal cancer * UICC Stage II/III*	5/11	45.5%
Early rectal cancer * UICC Stage I*	6/11	54.5%
Treatment types		
Primary resection	3/11	27.3%
Neoadjuvant chemoradiotherapy (nCRT)	5/11	45.5%
Neoadjuvant chemotherapy (nCTx)	3/11	27.3%
Definitive chemoradiotherapy (dCRT)	3/11	27.3%
Adjuvant chemotherapy (adCTx)	4/11	36.4%
Adjuvant radiotherapy	1/11	9.1%

All included reports were assessed regarding their methodological quality [17]. Of these, 5/9 reports fulfilled the specified criteria for good quality. Significance of the remaining three case reports was limited due to short follow-up (< 12 months) or fair (one case) due to inadequate reporting of follow-up, outcomes, and toxicity.

## Discussion

We here provide the first systematic overview of curative-intended treatments for synchronous EC and RC. The small number of reported cases highlights the rarity of this coincidence, although a relevant number of undiscovered or unreported cases have to be assumed. Colonoscopy is not routinely performed in all EC-patients consistently, and early-stage RC might remain asymptomatic, and, hence, undiscovered because the prognostic determinant might be EC rather than limited RC. Furthermore, the increasing availability of FDG-PET/CT for staging might lead to a rising number of incidental asymptomatic second primary cancers. For example, RC was diagnosed through FDG-PET/CT-staging in the two herein reported cases. Different reports indicate that a relevant proportion (10–17%) of patients with primary EC simultaneously presents with multiple primary tumors. Mostly, second primary head/neck cancers were reported. Less frequently, other gastrointestinal cancers, such as RC (3%), occurred [25–29]. Primary resection can be offered, but data is heterogeneous regarding the outcome in the presence of a secondary colorectal neoplasia [30, 31]. Nevertheless, the herein reported cases have often been considered being too advanced for primary surgery or a simultaneous extended resection of both foci seemed to be too stressing for the patients. A multimodal approach was consequently performed and primary resection of both tumors was only realized within a minority of the patients. Most of the published cases occurred in Japan or India, underlining the high incidence and expertise in management of gastrointestinal cancer in Asia [32].

A chronic exposure to alcohol or nicotine might increase the risk for synchronous EC and RC, as it is reported for head/neck-cancer patients, presenting similar risk factors, being prone for second primary cancer [33]. As all cases were male and of increased age (> 60 years), mostly presenting with ESCC (8/9), chronic exposure to noxious substances might be assumed. Both risk factors and, furthermore, obesity were present in our two patients. All these are known independent risk factors for colorectal cancer [1]. However, this information was not available for the other reported cases, and the small sample size does neither allow for testing nor concluding any attribution.

To date, there is no consensus on optimal treatment strategies or choice of systemic treatment agents for synchronous cancers. Chemotherapy was in particular consisting of pyrimidine-based agents. We also preferred 5-FU-based concomitant chemotherapy over the CROSS-protocol, as it appeared to be effective for rectal adenocarcinoma histology simultaneously. Besides, this protocol ensured effective salvage options, as ICI was approved in Germany for second-line treatment after 5-FU-based first-line treatment failure back then. Yoshii et al. [34] reported a series of patients receiving an even more intense concomitant chemotherapy (FOLFOX) with promising results. As the underlying PRODIGE-5-Trial failed to demonstrate superiority of FOLFOX compared to 5-FU/cisplatin [35], we preferred the CALGB-9871 chemotherapy protocol [36]. But, given the current trials for TNT, which mostly incorporate FOLFOX [5, 7–9], and the PROSPECT-data showing respectable tumor regression in low/intermediate risk RC after sole neoadjuvant chemotherapy [37], FOLFOX might be an effective option to hit both cancers. No RT for RC was performed in the patients reported by Yoshii et al., and one patient achieved CR of RC after six courses FOLFOX. Nevertheless, CR after FOLFOX alone is a rare event in RC. If NOM was intended, a local treatment intensification by pelvic RT and/or rectal brachytherapy might be beneficial given the encouraging results of current trials on organ preservation approaches [9, 38–41]. There was no case with primary treatment intended to perform an organ preservation approach of RC. Intended NOM for RC would probably require specific local treatment, such as local percutaneous radiotherapy and/or brachytherapy (depending on RC stage). However, our case 2 illustrates that sequential intensive local treatment RC might not be realizable as planned due to toxicity from preceding treatment of EC. Considering the likely worse prognosis of (locally advanced) EC, we feel that the local therapy of RC should not be prioritized with the goal of NOM, in order to avoid compromising the treatment of the EC. Consistently, treatment of EC was prioritized from all other authors, except in cases with severe stenosis of the RC, where immediate resection was performed.

The most reported treatments seemed to be effective and well tolerated. However, no preferred regime could be derived even from this sample. Of note, our case 1 and the case reported by Utsunomiya et al. 2013 [23] demonstrate the successful curative-intended definitive treatment of an initially oligometastatic synchronous EC/RC.

Overall reporting quality of the case reports was appropriate according to the criteria proposed by Murad et al. [17], except of one case (fair quality). The significance was mostly limited to relatively short follow-up, probably overestimating “real-world” PFS and OS (Fig. 4). One major issue is the inherent risk of bias in case reports, such as selection bias, as these reports often focus on exceptional or atypical cases, potentially overrepresenting successful outcomes. Additionally, this analysis is prone to publication bias, as cases with positive or notable results are more likely to be reported. Another critical limitation is the short follow-up period in these case reports, which may not allow for a comprehensive assessment of long-term outcomes, recurrence rates, or late-onset side effects of the combined therapy. These factors undermine the generalizability and robustness of conclusions drawn from such data. In particular, this analysis allows only limited conclusions regarding the effectiveness in tumor control and survival. For example, our case 2 represents one of the few instances of therapy failure with an extended follow-up period, which highlights the limitations previously mentioned, such as the underrepresentation of negative outcomes and the lack of long-term data in most case reports. Hence, we encourage future structured reporting of clinical patient data in order to derive substantiated implications for clinical routine. However, this is the first comprehensive analysis on this rare combination of therapies, providing crucial insights into the feasibility and acute toxicity of the combined multimodal treatment. This review offers valuable information that can guide future clinical decisions and help improve the understanding of the risks and benefits associated with simultaneous treatment of EC and RC.

## Conclusion

Despite the presumably relevant proportion of patients with EC presenting with synchronous RC, there is no reliable data on optimal management available up to now. The reported cases demonstrate encouraging results for multimodal treatments. Especially neoadjuvant CRT, incorporating 5-FU-based protocols, seems to be a promising approach, occasionally allowing for definitive treatment/NOM. Nevertheless, these findings cannot simply be generalized, and generating high-quality evidence with prospective clinical trials will be hardly realizable.

## Supplementary Information

Below is the link to the electronic supplementary material.

Supplementary file1 (DOCX 33 KB)

## Data Availability

No datasets were generated or analyzed during the current study.

## Abbreviations

*5-FU*: 5-Fluorouracil
*CR*: Complete response
*CRT*: Chemoradiotherapy
*CTCAE*: Common Terminology Criteria for Adverse Events
*DWI*: Diffusion-weighted imaging
*EAC*: Esophageal adenocarcinoma
*EC*: Esophageal cancer
*ESCC*: Esophageal squamous cell cancer
*FDG-PET/CT*: Fluorodeoxyglucose positron emission tomography/computed tomography
*ICI*: Immune-checkpoint inhibition
*MeSH*: Medical subject headings
*MRI*: Magnetic resonance imaging
*MSI-H*: Micro-satellite instability high
*NED*: No evidence of disease
*NOM*: Non-operative management
*NR*: No response
*PD*: Progressive disease
*PFS*: Progression-free survival
*PICO*: Population, Intervention, Comparison, Outcome
*PR*: Partial response
*PSA*: Prostate-specific membrane antigen
*RAMIE*: Robot-assisted minimally invasive esophagectomy
*RC*: Rectal cancer
*REUS*: Endorectal ultrasound
*SD*: Stable disease
*SFD*: Single fraction dose
*SOC*: Standard of care
*TAD*: Total absorbed dose
