# Supplementary material for: Curative-Intended Management of Synchronous Esophageal and Rectal Cancer—A Systematic Literature Review
Source: J Gastrointest Cancer. 2025 Jan 13;56(1):41. doi: 10.1007/s12029-025-01170-7 (PMC11725541; doi:10.1007/s12029-025-01170-7)
Supplement: Supplementary file 1 — Supplementary file1 (DOCX 33 KB) [file 12029_2025_1170_MOESM1_ESM.docx]

## *Supplementary Content*

# PICO Model

Table 1 Inclusion and exclusion criteria based on a PICO model (Population, Intervention, Comparison, Outcome).

| PICO | Inclusion criteria | Exclusion criteria |
| --- | --- | --- |
| Patient | Patients with simultaneous diagnosis of invasive esophageal cancer and rectal cancer.  Additional synchronous cancer was allowed. | Patients with precancerous conditions or carcinoma in situ  Primary prevention |
| Intervention | Treatment with curative intend |  |
| Comparison | None |  |
| Outcome | Mortality (Survival)  Morbidity (progression-/disease-free interval, tumor response)  Toxicity and adverse events | Reports lacking sufficient individual data on characteristics, treatment or follow-up information |
| Others | Full publication available | Gray literature (e.g. conference articles, abstracts, letters, ongoing studies, unpublished literature)  Full text not available |

# Search string

## Pubmed

(("rectal neoplasms"[MeSH Terms] OR ("rectal"[Title/Abstract] AND "neoplasms"[Title/Abstract]) OR "rectal neoplasms"[Title/Abstract] OR ("rectum"[Title/Abstract] AND "cancer"[Title/Abstract]) OR "rectum cancer"[Title/Abstract])

AND

("oesophageal cancer"[Title/Abstract] OR "esophageal neoplasms"[MeSH Terms] OR ("esophageal"[Title/Abstract] AND "neoplasms"[Title/Abstract]) OR "esophageal neoplasms"[Title/Abstract] OR ("esophageal"[Title/Abstract] AND "cancer"[Title/Abstract]) OR "esophageal cancer"[Title/Abstract]))

OR

(("rectum"[MeSH Terms] OR "rectum"[Title/Abstract] OR "rectums"[Title/Abstract] OR "rectal"[Title/Abstract] OR “rect*”[Title/Abstract] OR "colorectal"[Title/Abstract] OR "colorect*"[Title/Abstract])

AND

(("oesophagus"[Title/Abstract] OR "esophagus"[MeSH Terms] OR "esophagus"[Title/Abstract])

OR

("esophageal"[Title/Abstract] OR "esophagic"[Title/Abstract] OR "oesophagal"[Title/Abstract] OR "oesophageal"[Title/Abstract] OR "oesophagic"[Title/Abstract] OR “esophag*”[Title/Abstract] OR “oesophag*”[Title/Abstract] OR “esophagogastr*”[Title/Abstract] OR “oesophagogastr*”[Title/Abstract]))

AND

(("cancer s"[Title/Abstract] OR "cancerated"[Title/Abstract] OR "canceration"[Title/Abstract] OR "cancerization"[Title/Abstract] OR "cancerized"[Title/Abstract] OR "cancerous"[Title/Abstract] OR "neoplasms"[MeSH Terms] OR "neoplasms"[Title/Abstract] OR "cancer"[Title/Abstract] OR "cancers"[Title/Abstract])

OR

("neoplasm s"[Title/Abstract] OR "neoplasms"[MeSH Terms] OR "neoplasms"[Title/Abstract] OR "neoplasm"[Title/Abstract])

OR

("malign"[Title/Abstract] OR "malignance"[Title/Abstract] OR "malignances"[Title/Abstract] OR "malignant"[Title/Abstract] OR "malignants"[Title/Abstract] OR "malignities"[Title/Abstract] OR "malignity"[Title/Abstract] OR "malignization"[Title/Abstract] OR "malignized"[Title/Abstract] OR "maligns"[Title/Abstract] OR "neoplasms"[MeSH Terms] OR "neoplasms"[Title/Abstract] OR "malignancies"[Title/Abstract] OR "malignancy"[Title/Abstract])))

AND

(("synchronic"[Title/Abstract] OR "synchronically"[Title/Abstract] OR "synchronicities"[Title/Abstract] OR "synchronicity"[Title/Abstract] OR "synchronisation"[Title/Abstract] OR "synchronisations"[Title/Abstract] OR "synchronise"[Title/Abstract] OR "synchronised"[Title/Abstract] OR "synchroniser"[Title/Abstract] OR "synchronisers"[Title/Abstract] OR "synchronises"[Title/Abstract] OR "synchronising"[Title/Abstract] OR "synchronism"[Title/Abstract] OR "synchronisms"[Title/Abstract] OR "synchronization"[Title/Abstract] OR "synchronizations"[Title/Abstract] OR "synchronize"[Title/Abstract] OR "synchronized"[Title/Abstract] OR "synchronizer"[Title/Abstract] OR "synchronizers"[Title/Abstract] OR "synchronizes"[Title/Abstract] OR "synchronizing"[Title/Abstract] OR "synchronous"[Title/Abstract] OR "synchronously"[Title/Abstract] OR "synchronous*"[Title/Abstract])

OR

(“concomitance"[Title/Abstract] OR "concomitant"[Title/Abstract] OR "concomitants"[Title/Abstract] OR "concomit*"[Title/Abstract])

OR

("coincide"[Title/Abstract] OR "coincided"[Title/Abstract] OR "coincident"[Title/Abstract] OR "coincidently"[Title/Abstract] OR "coincides"[Title/Abstract] OR "coinciding"[Title/Abstract] OR "coinci*"[Title/Abstract])

OR

("coexist"[Title/Abstract] OR "coexistance"[Title/Abstract] OR "coexistant"[Title/Abstract] OR "coexisted"[Title/Abstract] OR "coexistence"[Title/Abstract] OR "coexistences"[Title/Abstract] OR "coexistent"[Title/Abstract] OR "coexisting"[Title/Abstract] OR "coexists"[Title/Abstract] OR "coexist*"[Title/Abstract])

OR

("concurrent"[Title/Abstract] OR "concurrently"[Title/Abstract] OR "concurrents"[Title/Abstract] OR "concurrent*"[Title/Abstract])

OR

("simultaneous"[Title/Abstract] OR "simultaneously"[Title/Abstract] OR "simultaneous*"[Title/Abstract]))

## OVID Medline

((exp "rectal neoplasms"/ OR (rectal.tw. AND neoplasms.tw.) OR "rectal neoplasms".tw. OR (rectum.tw. AND cancer.tw.) OR "rectum cancer".tw.)
AND
("oesophageal cancer".tw. OR exp "esophageal neoplasms"/ OR (esophageal.tw. AND neoplasms.tw.) OR "esophageal neoplasms".tw. OR (esophageal.tw. AND cancer.tw.) OR "esophageal cancer".tw.))
OR
((exp rectum/ OR rectum.tw. OR rectums.tw. OR rectal.tw. OR rect*.tw. OR colorectal.tw. OR colorect*.tw.)
AND
((oesophagus.tw. OR exp esophagus/ OR esophagus.tw.)
OR
(esophageal.tw. OR esophagic.tw. OR oesophagal.tw. OR oesophageal.tw. OR oesophagic.tw. OR esophag*.tw. OR oesophag*.tw. OR esophagogastr*.tw. OR oesophagogastr*.tw.))
AND
(("cancer s".tw. OR cancerated.tw. OR canceration.tw. OR cancerization.tw. OR cancerized.tw. OR cancerous.tw. OR exp neoplasms/ OR neoplasms.tw. OR cancer.tw. OR cancers.tw.)
OR
("neoplasm s".tw. OR exp neoplasms/ OR neoplasms.tw. OR neoplasm.tw.)
OR
(malign.tw. OR malignance.tw. OR malignances.tw. OR malignant.tw. OR malignants.tw. OR malignities.tw. OR malignity.tw. OR malignization.tw. OR malignized.tw. OR maligns.tw. OR exp neoplasms/ OR neoplasms.tw. OR malignancies.tw. OR malignancy.tw.)))
AND
((synchronic.tw. OR synchronically.tw. OR synchronicities.tw. OR synchronicity.tw. OR synchronisation.tw. OR synchronisations.tw. OR synchronise.tw. OR synchronised.tw. OR synchroniser.tw. OR synchronisers.tw. OR synchronises.tw. OR synchronising.tw. OR synchronism.tw. OR synchronisms.tw. OR synchronization.tw. OR synchronizations.tw. OR synchronize.tw. OR synchronized.tw. OR synchronizer.tw. OR synchronizers.tw. OR synchronizes.tw. OR synchronizing.tw. OR synchronous.tw. OR synchronously.tw. OR synchronous*.tw.)
OR
(concomitance.tw. OR concomitant.tw. OR concomitants.tw. OR concomit*.tw.)
OR
(coincide.tw. OR coincided.tw. OR coincident.tw. OR coincidently.tw. OR coincides.tw. OR coinciding.tw. OR coinci*.tw.)
OR
(coexist.tw. OR coexistance.tw. OR coexistant.tw. OR coexisted.tw. OR coexistence.tw. OR coexistences.tw. OR coexistent.tw. OR coexisting.tw. OR coexists.tw. OR coexist*.tw.)
OR
(concurrent.tw. OR concurrently.tw. OR concurrents.tw. OR concurrent*.tw.)
OR
(simultaneous.tw. OR simultaneously.tw. OR simultaneous*.tw.))

## Cochrane

(([mh "rectal neoplasms"] OR (rectal:ti,ab AND neoplasms:ti,ab) OR "rectal neoplasms":ti,ab OR (rectum:ti,ab AND cancer:ti,ab) OR "rectum cancer":ti,ab)
AND
("oesophageal cancer":ti,ab OR [mh "esophageal neoplasms"] OR (esophageal:ti,ab AND neoplasms:ti,ab) OR "esophageal neoplasms":ti,ab OR (esophageal:ti,ab AND cancer:ti,ab) OR "esophageal cancer":ti,ab))
OR
(([mh rectum] OR rectum:ti,ab OR rectums:ti,ab OR rectal:ti,ab OR rect*:ti,ab OR colorectal:ti,ab OR colorect*:ti,ab)
AND
((oesophagus:ti,ab OR [mh esophagus] OR esophagus:ti,ab)
OR
(esophageal:ti,ab OR esophagic:ti,ab OR oesophagal:ti,ab OR oesophageal:ti,ab OR oesophagic:ti,ab OR esophag*:ti,ab OR oesophag*:ti,ab OR esophagogastr*:ti,ab OR oesophagogastr*:ti,ab))
AND
(("cancer s":ti,ab OR cancerated:ti,ab OR canceration:ti,ab OR cancerization:ti,ab OR cancerized:ti,ab OR cancerous:ti,ab OR [mh neoplasms] OR neoplasms:ti,ab OR cancer:ti,ab OR cancers:ti,ab)
OR
("neoplasm s":ti,ab OR [mh neoplasms] OR neoplasms:ti,ab OR neoplasm:ti,ab)
OR
(malign:ti,ab OR malignance:ti,ab OR malignances:ti,ab OR malignant:ti,ab OR malignants:ti,ab OR malignities:ti,ab OR malignity:ti,ab OR malignization:ti,ab OR malignized:ti,ab OR maligns:ti,ab OR [mh neoplasms] OR neoplasms:ti,ab OR malignancies:ti,ab OR malignancy:ti,ab)))
AND
((synchronic:ti,ab OR synchronically:ti,ab OR synchronicities:ti,ab OR synchronicity:ti,ab OR synchronisation:ti,ab OR synchronisations:ti,ab OR synchronise:ti,ab OR synchronised:ti,ab OR synchroniser:ti,ab OR synchronisers:ti,ab OR synchronises:ti,ab OR synchronising:ti,ab OR synchronism:ti,ab OR synchronisms:ti,ab OR synchronization:ti,ab OR synchronizations:ti,ab OR synchronize:ti,ab OR synchronized:ti,ab OR synchronizer:ti,ab OR synchronizers:ti,ab OR synchronizes:ti,ab OR synchronizing:ti,ab OR synchronous:ti,ab OR synchronously:ti,ab OR synchronous*:ti,ab)
OR
(concomitance:ti,ab OR concomitant:ti,ab OR concomitants:ti,ab OR concomit*:ti,ab)
OR
(coincide:ti,ab OR coincided:ti,ab OR coincident:ti,ab OR coincidently:ti,ab OR coincides:ti,ab OR coinciding:ti,ab OR coinci*:ti,ab)
OR
(coexist:ti,ab OR coexistance:ti,ab OR coexistant:ti,ab OR coexisted:ti,ab OR coexistence:ti,ab OR coexistences:ti,ab OR coexistent:ti,ab OR coexisting:ti,ab OR coexists:ti,ab OR coexist*:ti,ab)
OR
(concurrent:ti,ab OR concurrently:ti,ab OR concurrents:ti,ab OR concurrent*:ti,ab)
OR
(simultaneous:ti,ab OR simultaneously:ti,ab OR simultaneous*:ti,ab))

## Web of Science

(("rectal neoplasms" OR (rectal AND neoplasms) OR "rectal neoplasms" OR (rectum AND cancer) OR "rectum cancer")
AND
("oesophageal cancer" OR "esophageal neoplasms" OR (esophageal AND neoplasms) OR "esophageal neoplasms" OR (esophageal AND cancer) OR "esophageal cancer"))
OR
((rectum OR rectum OR rectums OR rectal OR rect* OR colorectal OR colorect*)
AND
((oesophagus OR esophagus OR esophagus)
OR
(esophageal OR esophagic OR oesophagal OR oesophageal OR oesophagic OR esophag* OR oesophag* OR esophagogastr* OR oesophagogastr*))
AND
(("cancer s" OR cancerated OR canceration OR cancerization OR cancerized OR cancerous OR neoplasms OR neoplasms OR cancer OR cancers)
OR
("neoplasm s" OR neoplasms OR neoplasms OR neoplasm)
OR
(malign OR malignance OR malignances OR malignant OR malignants OR malignities OR malignity OR malignization OR malignized OR maligns OR neoplasms OR neoplasms OR malignancies OR malignancy)))
AND
((synchronic OR synchronically OR synchronicities OR synchronicity OR synchronisation OR synchronisations OR synchronise OR synchronised OR synchroniser OR synchronisers OR synchronises OR synchronising OR synchronism OR synchronisms OR synchronization OR synchronizations OR synchronize OR synchronized OR synchronizer OR synchronizers OR synchronizes OR synchronizing OR synchronous OR synchronously OR synchronous*)
OR
(concomitance OR concomitant OR concomitants OR concomit*)
OR
(coincide OR coincided OR coincident OR coincidently OR coincides OR coinciding OR coinci*)
OR
(coexist OR coexistance OR coexistant OR coexisted OR coexistence OR coexistences OR coexistent OR coexisting OR coexists OR coexist*)
OR
(concurrent OR concurrently OR concurrents OR concurrent*)
OR
(simultaneous OR simultaneously OR simultaneous*))

## SCOPUS

((INDEXTERMS("rectal neoplasms") OR (TITLE-ABS(rectal) AND TITLE-ABS(neoplasms)) OR TITLE-ABS("rectal neoplasms") OR (TITLE-ABS(rectum) AND TITLE-ABS(cancer)) OR TITLE-ABS("rectum cancer"))
AND
(TITLE-ABS("oesophageal cancer") OR INDEXTERMS("esophageal neoplasms") OR (TITLE-ABS(esophageal) AND TITLE-ABS(neoplasms)) OR TITLE-ABS("esophageal neoplasms") OR (TITLE-ABS(esophageal) AND TITLE-ABS(cancer)) OR TITLE-ABS("esophageal cancer")))
OR
((INDEXTERMS(rectum) OR TITLE-ABS(rectum) OR TITLE-ABS(rectums) OR TITLE-ABS(rectal) OR TITLE-ABS(rect*) OR TITLE-ABS(colorectal) OR TITLE-ABS(colorect*))
AND
((TITLE-ABS(oesophagus) OR INDEXTERMS(esophagus) OR TITLE-ABS(esophagus))
OR
(TITLE-ABS(esophageal) OR TITLE-ABS(esophagic) OR TITLE-ABS(oesophagal) OR TITLE-ABS(oesophageal) OR TITLE-ABS(oesophagic) OR TITLE-ABS(esophag*) OR TITLE-ABS(oesophag*) OR TITLE-ABS(esophagogastr*) OR TITLE-ABS(oesophagogastr*)))
AND
((TITLE-ABS("cancer s") OR TITLE-ABS(cancerated) OR TITLE-ABS(canceration) OR TITLE-ABS(cancerization) OR TITLE-ABS(cancerized) OR TITLE-ABS(cancerous) OR INDEXTERMS(neoplasms) OR TITLE-ABS(neoplasms) OR TITLE-ABS(cancer) OR TITLE-ABS(cancers))
OR
(TITLE-ABS("neoplasm s") OR INDEXTERMS(neoplasms) OR TITLE-ABS(neoplasms) OR TITLE-ABS(neoplasm))
OR
(TITLE-ABS(malign) OR TITLE-ABS(malignance) OR TITLE-ABS(malignances) OR TITLE-ABS(malignant) OR TITLE-ABS(malignants) OR TITLE-ABS(malignities) OR TITLE-ABS(malignity) OR TITLE-ABS(malignization) OR TITLE-ABS(malignized) OR TITLE-ABS(maligns) OR INDEXTERMS(neoplasms) OR TITLE-ABS(neoplasms) OR TITLE-ABS(malignancies) OR TITLE-ABS(malignancy))))
AND
((TITLE-ABS(synchronic) OR TITLE-ABS(synchronically) OR TITLE-ABS(synchronicities) OR TITLE-ABS(synchronicity) OR TITLE-ABS(synchronisation) OR TITLE-ABS(synchronisations) OR TITLE-ABS(synchronise) OR TITLE-ABS(synchronised) OR TITLE-ABS(synchroniser) OR TITLE-ABS(synchronisers) OR TITLE-ABS(synchronises) OR TITLE-ABS(synchronising) OR TITLE-ABS(synchronism) OR TITLE-ABS(synchronisms) OR TITLE-ABS(synchronization) OR TITLE-ABS(synchronizations) OR TITLE-ABS(synchronize) OR TITLE-ABS(synchronized) OR TITLE-ABS(synchronizer) OR TITLE-ABS(synchronizers) OR TITLE-ABS(synchronizes) OR TITLE-ABS(synchronizing) OR TITLE-ABS(synchronous) OR TITLE-ABS(synchronously) OR TITLE-ABS(synchronous*))
OR
(TITLE-ABS(concomitance) OR TITLE-ABS(concomitant) OR TITLE-ABS(concomitants) OR TITLE-ABS(concomit*))
OR
(TITLE-ABS(coincide) OR TITLE-ABS(coincided) OR TITLE-ABS(coincident) OR TITLE-ABS(coincidently) OR TITLE-ABS(coincides) OR TITLE-ABS(coinciding) OR TITLE-ABS(coinci*))
OR
(TITLE-ABS(coexist) OR TITLE-ABS(coexistance) OR TITLE-ABS(coexistant) OR TITLE-ABS(coexisted) OR TITLE-ABS(coexistence) OR TITLE-ABS(coexistences) OR TITLE-ABS(coexistent) OR TITLE-ABS(coexisting) OR TITLE-ABS(coexists) OR TITLE-ABS(coexist*))
OR
(TITLE-ABS(concurrent) OR TITLE-ABS(concurrently) OR TITLE-ABS(concurrents) OR TITLE-ABS(concurrent*))
OR
(TITLE-ABS(simultaneous) OR TITLE-ABS(simultaneously) OR TITLE-ABS(simultaneous*)))

# Studies excluded after full-text screening:

| **Excluded studies** | **Reason for exclusion** |
| --- | --- |
| Eskarous et al. 2020 [1] | Treated with palliative intention. |
| Kagei et al. 2002 [2] | No sufficient individual patient data regarding tumor characteristics, treatment and follow-up reported. |
| Nakamura et al. 1988 [3] | Treated with palliative intention. |
| Solecki et al. 2004 [4] | Treated with palliative intention. |
| Sönmez et al. 2021 [5] | Report lacked sufficient data on tumor characteristics and follow-up |
| Takeuchi et al. 2015 [6] | No sufficient individual patient data regarding tumor characteristics, treatment and follow-up reported. |

**References:**

1. Eskarous, H., et al., *Synchronous Non-Hereditary Esophageal and Colorectal Cancer in a Young, Healthy Male Patient.* American Journal of Gastroenterology, 2020. **115**.

2. Kagei, K., et al., *Efficacy of intense screening and treatment for synchronous second primary cancers in patients with esophageal cancer.* Jpn J Clin Oncol, 2002. **32**(4).

3. Nakamura, K., et al., *A case of synchronous rectum cancer and primary oat cell cancer of esophagus.* Nippon Shokakibyo Gakkai Zasshi, 1988. **85**(5).

4. Solecki, R., et al., *Bifocal esophageal and rectal cancer palliatively treated with argon plasma coagulation.* Surg Endosc, 2004. **18**(2).

5. Sönmez, G.M., et al., *Multiple primary synchronous gastric, esophageal, and rectal cancer and isolated esophageal metastasis from rectal cancer: Case report.* Journal of Oncological Science, 2021. **7**(3).

6. Takeuchi, D., et al., *Prevalence and management of colorectal neoplasia in surgically treated esophageal cancer patients.* Int J Surg, 2015. **17**.
